# Validation of the GRACE score risk in our population

**DOI:** 10.1186/cc12173

**Published:** 2013-03-19

**Authors:** L Sayagues, J Sieira, E Abbu, J Chico, C Pena, J Gonzalez Juantey

**Affiliations:** 1 HULA, Lugo, Spain; 2 CHUS, Santiago de Compostela, Spain; 3 CHUVI, Vigo, Spain

## Introduction

The Global Registry of Acute Coronary Events (GRACE) risk score provides an estimation of the probability of death within 6 months of hospital discharge in patients with acute coronary syndrome (ACS). Our aim was to assess the validity of this risk score in our contemporary cohort of patients admitted to our third-level hospital.

## Methods

The study involved 1,185 consecutive patients with ACS evaluated between February 2004 and February 2009. Their virtual status was determined 6 months after hospital discharge and the validity of the GRACE risk score was evaluated.

## Results

In total, 450 (38.8%) patients were admitted for STEMI and 725 (61.2%) for NSTEMI. Percutaneous revascularisation was performed in 846 (71.5%). The median GRACE risk score was 121 (interquartile range 96/144). Mortality after discharge was 4.4%. The calibration of the GRACE score was Hosmer-Lemeshow *P >*0.2 and its discriminatory capacity was excellent. Area under the ROC curve was 0.86, 95% CI 0.807 to 0.916, in all patients. See Table 1.

**Table 1:** 

GRACE risk	Total	STEMI	NSTEMI
Low	270 (22.7)	177 (38.3)	93 (12.8)
Medium	334 (28.2)	141 (30.7)	193 (26.7)
High	580 (49)	142 (30.9)	438 (60.5)

## Conclusion

The GRACE risk score for predicting death within 6 months of hospital discharge was validated and can be used in patients with ACS. It would be perfect in the future to include the GRACE risk score in the medical records of this type of patients. Also it would be very interesting to validate this in a multicentric study.

